# Autologous Serum Skin Test as a Diagnostic Aid in Chronic Idiopathic Urticaria

**DOI:** 10.1155/2013/291524

**Published:** 2013-04-18

**Authors:** Hayder R. Al-Hamamy, Ammar F. Hameed, Asaad S. Abdulhadi

**Affiliations:** ^1^Department of Dermatology and Venereology, College of Medicine, University of Baghdad, Medical Collection Office, P.O. Box 61211, Baghdad 12114, Iraq; ^2^Department of Dermatology and Venereology, Baghdad Teaching Hospital, Baghdad, Iraq

## Abstract

*Background*. Chronic urticaria is defined as urticaria persisting daily for more than six weeks. A significant number of patients had autoimmune basis where autologous serum skin test is widely used for detection of chronic autoimmune urticaria. *Objectives*. To estimate the frequency of autoimmune urticarial in Iraqi patients utilizing the autologous serum skin test and to evaluate its results with the variable clinical features of chronic idiopathic urticaria. *Methods*. In this prospective study, 54 patients with chronic idiopathic urticaria were investigated with autologous serum skin test where its results were examined with the different clinical parameters of chronic autoimmune urticaria. *Results*. Twenty two patients (40.7%) out of 54 patients with chronic idiopathic urticarial had positive autologous serum skin test. Statistical analysis of the clinical variables did not show a significant difference between patients with positive and negative autologous serum skin test except for the distribution of wheals on the face and extremities which was significantly associated with positive autologous serum skin test results (*P* value 0.004). *Conclusion*. Autologous serum skin test is a simple, office-based test for detecting chronic autoimmune urticaria patients who have no distinctive clinical features differentiating them from chronic idiopathic urticaria patients.

## 1. Introduction

Chronic urticaria is defined as urticaria persisting daily or almost daily for more than six weeks [1]. Although in many patients with chronic urticaria the disease remains idiopathic, in the last years, a significant number of patients with chronic urticaria have been proved to have autoimmune causes for their urticaria [2].

The autoimmune subgroup constitutes about 45% of patients previously diagnosed as having chronic idiopathic urticaria (CIU), and it is associated with the IgG anti-IgE receptor alpha subunit in 35–40% of the patients or IgG anti-IgE in additional 5–10%. These autoantibodies have been shown to activate blood basophils and cutaneous mast cells *in vitro* [3]. The presence of these autoantibodies may be important clinically in a group of severely affected, treatment-resistant patients, where immunomodulatory treatments may be valuable [2]. Clinical examination of the patient with CIU is generally unhelpful in distinguishing autoimmune from nonautoimmune patients [4]. 

Autologous serum skin test (ASST) is a simple *in vivo* clinical test for the detection of basophil histamine-releasing activity. Sabroe et al. found that ASST has a sensitivity of approximately 70% and a specificity of 80%, and it may be used as a reasonably predictive clinical test to indicate the presence of functional circulating autoantibodies [5]. The present work studied the results of ASST in CIU patients and its correlation with the clinical features of CIU patients.

## 2. Patients and Methods

This prospective study was conducted in Department of Dermatology and Venereology in Baghdad Teaching Hospital during the period from November 2009 to March 2011. The number of examined patients was 54. All patients have active urticaria at time of presentation and the current attack started at least six week before the test; children younger than 15 years of age and pregnant women were excluded from the study.

Full history was taken from each patient including age, sex, duration of the disease, severity, duration of current attack, and age at onset and a severity score was made as shown in Table 1.

Also we interrogated about family history of urticaria, personal and family history atopy, and personal and family history of autoimmune diseases (like rheumatoid arthritis, lupus erythematosus, vitiligo, and thyroid diseases).

The nature of the study was clarified to the patients where they gave their written informed consent and the study was approved by the Ethical Committee of Iraqi Board for Medical Specializations.

All patients had stopped any systemic treatment at least 3 days before the test and only topical antipruritics like calamine lotion were allowed if the itching was severe. The autologous serum skin test (ASST) was performed by drawing 2 cubic centimeters of venous blood and storing it in a plane tube (not heparinized) and allowing it to clot at room temperature; after that the serum is separated by centrifuging the blood in a centrifuge for 10 minutes at 2000 RPM (revolution per minute). A volume of 0.05 mL of the patient's serum is injected intradermally on volar aspect of the patient's forearm at site not affected by a wheal in the last 24 hours; at the same time a control test is done by injecting the same volume of normal saline at least 5 centimeters away from the serum injection site; then we wait for 30 minutes to see the response. The test is considered positive and the patient is said to have chronic autoimmune urticaria (CAU) if there was a wheal and flare at the tested site (serum injection site) of a diameter (measured by taking the mean of the largest two diameters) at least 1.5 millimeters more than the wheal and flare induced at the control site (if any) [6].

Descriptive statistics were done by using scientific calculator. Analytic statistics were done by Chi-square. *P* value less than 0.05 was considered to be statistically significant. 

## 3. Results

Fifty four patients with CIU enrolled in this study; their ages ranged between 16 and 63 years with a mean age ± SD of 32 ± 11.7 years. patients were divided into five age groups (Table 2). Statistically there was no age group more susceptible to demonstrate antihistamine-releasing autoantibodies (*P* value 0.536).

Our study showed that 22 (40.7%) out of 54 patients with CIU were positive for the ASST.

Of total 54 patients, 37 were women and 17 men. The sex of the studied population did not affect the positivity of ASST (*P* value 0.966).

The severity score was divided into three groups: mild (≥5), moderate (6–10), and severe (>10) and their relation to the positivity of ASST was not significant (*P* value 0.406) (Table 2).

The mean disease duration ±SD was 32.19 ± 56.62 months. According to the duration of disease, the patients were divided into 6 groups where they did not influence the positivity of ASST (*P* value 0.578) (Table 2).

The frequency of attacks per week was distributed into three groups and there was no relation between the frequency of attacks and the positivity of ASST (*P* value 0.601) (Table 2).

There was no relation between results of ASST and the following parameters: presence of angioedema (*P*-value 0.641), presence of family history of urticaria (*P*-value 0.809), presence of family and/or personal history of atopy (*P* value 0.825), and presence of family and/or personal history of autoimmune diseases like thyroid disease, diabetes mellitus, vitiligo, and rheumatoid (*P* value 0.079).

Only the distribution of wheals on the body showed significant association with the positivity of ASST. The ASST result was significantly associated with the distribution on the face and extremities not the generalized urticarial rash (*P*-value 0.004) (Table 2).

## 4. Discussion

Chronic urticaria is a highly disabling disorder; recognition of chronic autoimmune urticaria (CAU) as a distinct subgroup of CIU during the last decades has facilitated better understanding of unremitting CIU [7]. These patients have histamine-releasing autoantibodies and they usually need high doses of antihistamines and/or systemic corticosteroids during acute exacerbations where the use of immunomodulatory drugs is not justified in CIU except in antihistamine refractory chronic urticaria cases [7]. 

A positive ASST has been associated with prolonged disease that is poorly responsive to routine therapy [7]. One important advantage of testing is to promote more tailored prognostic counseling and the earlier use of immunosuppressive drugs.

The basophil histamine release assay is currently the gold standard for detecting functional autoantibodies in the serum of patients with chronic urticaria. However, this bioassay is difficult to standardize because it requires fresh basophils from healthy donors and it is also time consuming where it remains restricted to scientific research. 

Hence, ASST is considered as a bedside clinical test which can detect the presence of autoimmunity in patients with CIU [8]. 

Asero et al. found that out of 78 patients with CIU, 35% had a positive ASST, but only 25% were positive for histamine-releasing activity against donor basophils [9]. 

Depending on the method of antibody detection, subsequent studies reported that prevalence of ASST positivity in patients of chronic urticaria varies from 35% to 58% [10–14].

In the present study 22 (40.7%) out of 54 patients with CIU were positive for the autologous serum skin test (ASST) which is comparable to the results of the previous studies.

In 1999, Sabroe et al. reported that ASST positive patients had more widespread lesions and significantly more severe pruritus and systemic symptoms [5]. Other studies also demonstrated a significant relation between CAU diagnosed by ASST and clinical parameters such as frequency of attacks, duration of individual episodes, duration of wheals, regional involvement, and being less responsive to conventional antihistamine therapy [10, 15, 16].

In the present study, different clinical parameters were studied to evaluate any proposed relationship between these parameters and the positivity of the ASST. 

These parameters are as follows: age of patients at presentation, age of patients at onset of urticaria, urticaria severity score, duration of the disease in months, duration of each wheal in hours, frequency of attacks per week, distribution of wheals, gender of the patients, interference of the disease with daily activities, presence of angioedema, interference of the disease with sleep, presence of family history of chronic urticaria, presence of personal and/or family history of atopy, and presence of personal and/or family history of autoimmune diseases. 

Statistical analysis of these patients did not show a significant difference between patients with positive and negative ASST regarding the above-mentioned parameters except for the distribution of wheals. In agreement with our observations, other studies found that patients with autoantibodies in their sera (CIU, who have positive ASST results) have no distinctive diagnostic clinical features that differentiate them from patients who do not have these antibodies (CIU, who have negative ASST results) [4, 17].

According to the present work, patients with CIU with urticarial wheals distributed on the face and extremities have more risk to demonstrate functional antihistamine autoantibody activity than those who have generalized urticarial distribution (*P*-value 0.004). This observation is not consistent with a study from India which reported no relation between regional involvements of CIU and the results of ASST [8]. 

In conclusion, ASST is simple, cost-effective test for detecting CAU patients who have no distinctive clinical features differentiating them from CIU patients. 

## Figures and Tables

**Table 1:** 

Frequency of attacks:	
(Days per week)	
1-2 days	1
3–5 days	2
>5 days	3
Duration of each wheal:	
<1 hour	1
1–6 hours	2
6–36 hours	3
Distribution of wheals:	
Face and extremities	1
All over the body	2
Interference with sleep:	
No	0
Yes	2
Interference with activities:	
No	0
Annoying	1
Abstinence from work	2
Associated angioedema:	
Eyelids, lips, or hands	1
Any 2 of above	2
All of them	3

The maximum score of severity is 15.

**Table 2:** The autologous serum skin test results in relation to different clinical parameters.

Variable	Positive ASST, *N* = 22 (40.7%)	Negative ASST, *N* = 32 (59.3%)	*P* value
Age (years)			
10–19	1 (1.9%)	6 (11.1%)	
20–29	8 (14.8%)	10 (18.5%)	0.536
30–39	7 (13.0%)	7 (13%)	
40–49	4 (7.4%)	4 (7.4%)	
≥50	2 (3.7%)	5 (9.3%)	
Sex			
Female	15 (27.8%)	22 (40.7%)	0.966
Male	7 (13.0%)	10 (18.5%)	
Score of severity			
≤5	2 (3.7%)	0 (0%)	
6–10	10 (18.5%)	15 (27.8)	0.406
≥10	10 (18.5%)	17 (31.5)	
Distribution of lesions			
All body	14 (25.9%)	30 (55.6%)	0.004
Face and extremities	8 (14.8%)	2 (3.7%)	
Duration of disease (months)			
≤12	13 (24.1%)	15 (27%)	
13–24	3 (5.6%)	6 (11.1%)	
25–36	1 (1.9%)	5 (9.3%)	0.578
37–48	0 (0%)	1 (1.9%)	
49–60	1 (1.9%)	1 (1.9%)	
>60	4 (7.4%)	4 (7.4%)	
